# Disparities in the use of colorectal cancer screening in a universally insured population during the COVID‐19 pandemic

**DOI:** 10.1002/cam4.6400

**Published:** 2023-08-29

**Authors:** Satish Munigala, Andrew J. Schoenfeld, Vivitha Mani, Amanda Banaag, Ada Umoh, Christian L. Coles, Tracey Perez Koehlmoos

**Affiliations:** ^1^ Center for Health Services Research Uniformed Services University of the Health Sciences Bethesda Maryland USA; ^2^ The Henry M. Jackson Foundation for the Advancement of Military Medicine, Inc. Bethesda Maryland USA; ^3^ Department of Orthopaedic Surgery and Center for Surgery and Public Health Brigham and Women's Hospital, Harvard Medical School Boston Massachusetts USA

**Keywords:** cancer prevention, colorectal cancer, epidemiology and prevention, screening

## Abstract

**Background:**

Despite the known efficacy of colorectal cancer (CRC) screening, the rates of individuals undergoing such testing have remained lower than target thresholds, even prior to the healthcare disruptions associated with the COVID‐19 pandemic. We evaluated the impact of the COVID‐19 pandemic on CRC screening within a nationally representative US population and assessed disparities in screening across racial/ethnic groups and socioeconomic (SES) strata.

**Methods:**

We performed a retrospective cross‐sectional study using all eligible TRICARE beneficiaries aged 45–64 years between FY 2018 and 2021. High‐risk individuals, those with a previous or current CRC diagnosis, and/or a personal/family history of colonic polyps, were excluded. The pre‐COVID‐19 period (September 1, 2018–March 31, 2020) was compared to the COVID‐19 period (April 1, 2020–September 30, 2021). Secondary analyses were performed, evaluating the interaction between the COVID‐19 time period, race, and our proxy for socioeconomic status.

**Results:**

During the study period, we identified 1,749,688 eligible individuals. Following the onset of the COVID‐19 pandemic, CRC screening overall decreased from 34% in the pre‐pandemic period to 30% following the onset of the pandemic (*p* < 0.001). This finding persisted even after adjusting for confounders in multivariable analysis (odds ratio [OR] for the pandemic timeframe: 0.79; 95% CI: 0.27, 0.31; *p* < 0.001). In the setting of SES, in the pandemic period, the odds of individuals from both Senior Enlisted (OR: 0.55; 95% CI: 0.54, 0.56) and Junior Enlisted sponsor ranks (OR: 0.27; 95% CI: 0.25, 0.30) were diminished as compared to Senior Officers.

**Conclusions and Relevance:**

We found a 21% reduction in the odds of CRC screening in the context of the COVID‐19 pandemic. Reductions in colonoscopies and other types of screening tests were not offset by changes in the use of at‐home tests such as Cologuard.

## 1 INTRODUCTION

Although the rates of colorectal cancer (CRC) diagnoses have diminished over the last four decades, this condition remains the second leading cause of cancer deaths in the United States, and the incidence is rising in individuals under age 50.^1^ The lifetime risk of CRC overall is 4%, and in 2019, there were approximately 1.4 million people living with this condition in the US.^2^ CRC is anticipated to cause over 50,000 deaths in 2023 alone and has the second highest treatment costs of any cancer at 23.7 billion USD per year.^3^ Screening for CRC has been shown to assist in early detection, which not only improves treatment efficacy and survival but may also reduce healthcare expenditures.^1^, ^3^

Despite the known efficacy of CRC screening, the rates of individuals undergoing such testing have remained lower than target thresholds, even prior to the healthcare disruptions precipitated by the COVID‐19 pandemic.^1^, ^2^, ^4^, ^5^ Disparities were already identified in the pre‐COVID‐19 window among minorities and those from lower socioeconomic strata (SES).^6^, ^7^, ^8^ These differences in access to screening as well as utilization may have been exacerbated further by disruptions resulting from COVID‐19, including the restricted performance of elective interventions such as colonoscopies.^4^, ^5^, ^9^, ^10^, ^11^, ^12^ At this time, reduced screening for CRC in the early stages of the pandemic is broadly acknowledged.^13^, ^14^, ^15^ However, specific impacts on underserved communities and changes in the use of screening modalities such as colonoscopy, home fecal immunochemical, or DNA testing kits have not been adequately explored. Previous work in this area may have been limited by geographic and sociodemographic restrictions in the populations under study, surveillance windows that only considered the early phases of the COVID‐19 pandemic, and cohorts that may not have been representative of the broader population eligible for CRC screening on the whole.

In this context, we sought to evaluate the influence of the COVID‐19 pandemic on the utilization of CRC screening among a representative US population, specifically TRICARE beneficiaries receiving services from the Military Health System (MHS). The use of TRICARE claims data make it possible to examine a large, universally insured cohort of individuals aged 45–64 who come from different racial and ethnic backgrounds and possess diverse socioeconomic, educational, and occupational characteristics.^16^, ^17^, ^18^, ^19^, ^20^, ^21^, ^22^ Use of TRICARE claims allowed us to determine individuals who received CRC screening regardless of their location, whether it was at home through mailed fecal testing kits or colonoscopy or other procedures performed in civilian healthcare settings (private sector care) or Department of Defense (DoD) hospitals (direct care).^18^, ^20^, ^21^ We believe that these attributes enable a more accurate assessment of the impact of the pandemic on CRC screening over a longer time frame and in a representative US cohort. Informed by prior studies,^6^, ^7^, ^12^ we hypothesized that CRC screening would decrease in the setting of the COVID‐19 pandemic and that disparities would be accentuated among racial minorities and individuals from lower SES.

## 2 METHODS

### 2.1 Data source and study cohort

This investigation employed TRICARE claims data that were derived from the Military Health System Data Repository (MDR). TRICARE, the health insurance plan of the DoD, provides healthcare coverage to active‐duty personnel, retirees, those medically retired with more than 30% disability, as well as dependents.^16^, ^17^, ^18^, ^19^, ^20^, ^21^, ^22^ TRICARE beneficiaries are able to access care through direct care institutions maintained by the DoD, as well as via civilian facilities where TRICARE operates as an insurance product within a fee‐for‐service setting (private‐sector care).^17^, ^18^, ^20^, ^21^, ^22^ As a result, TRICARE is among the largest healthcare insurance programs in the United States, with approximately 9.6 million participating beneficiaries.^16^, ^17^ The majority of these are civilian dependents or retirees, estimated at 80% of the covered population.^18^

The processes used to collect TRICARE claims as well as the means by which data are prepared and made accessible to researchers have been outlined in prior publications.^18^, ^19^, ^20^, ^22^ The MDR captures claims associated with both inpatient and outpatient encounters, irrespective of healthcare resource utilization, and captures both inpatient and outpatient services regardless of the site of service (e.g., DoD or civilian facility). Care delivered through the Veterans Administration (VA) system is not administered through TRICARE or captured in the MDR.^18^

**FIGURE 1:**
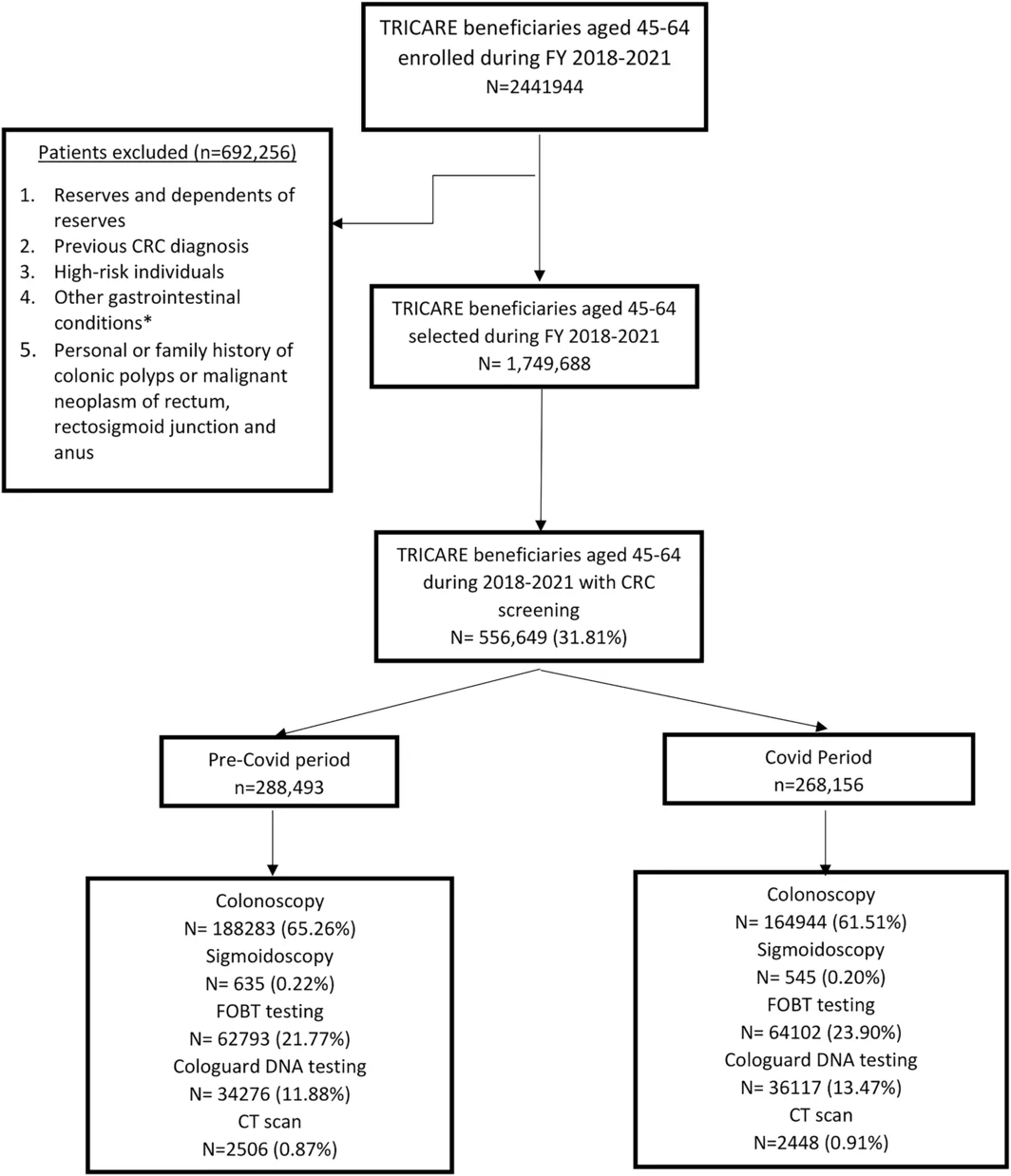
Study patients. CRC, colorectal cancer; FY, fiscal year. *prior diagnosis of diverticulitis, inflammatory bowel disease, Crohn's disease, ulcerative colitis or any benign/malignant lesions of colon.

We used the MDR records from September 1, 2018 to September 30, 2021, to identify TRICARE beneficiaries aged 45–64, at standard risk for CRC and eligible for screening. High‐risk individuals, as well as those with a previous or current diagnosis of CRC, related gastrointestinal disorders, and/or personal or family history of colonic polyps, were excluded (Figure 1). We abstracted data from the records of individuals eligible for inclusion to obtain age, race, biologic sex, sponsor rank, beneficiary category, branch of service affiliation, and healthcare service setting (direct or private sector). Race was recorded as documented in the MDR and based on individual self‐report. Our racial categories consisted of White, Black, Asian/Pacific Islander, American Indian/Alaskan Native, Other (e.g., mixed race/multiple race), and Missing. The MDR data does not specify ethnicity, so we were unable to identify those patients of Hispanic ethnicity. As such, White Hispanic patients are grouped in the White cohort, and Hispanic patients with Afro‐Caribbean ancestry would be categorized in the Black cohort. Sponsor rank was classified as Junior Enlisted, Senior Enlisted, Junior Officer, and Senior Officer. Aligned with previous research that supports the use of sponsor rank as a proxy for socioeconomic status,^18^, ^19^, ^20^, ^23^, ^24^ we considered enlisted individuals as representative of lower SES and compared these classes to Senior Officers as the referent.

CRC screening events were considered the first procedure performed within the study period based on Current Procedural Terminology and Healthcare Common Procedure Coding System codes (Table S1) for an eligible individual.

### 2.2 Statistical analysis

We compared the rates of CRC screening across two time‐windows^4^, ^5^, ^9^, ^10^, ^11^, ^12^: the pre‐COVID‐19 period (September 1, 2018–March 31, 2020) and the COVID‐19 period (April 1, 2020–September 30, 2021). Bivariate and multivariable logistic regression were used to assess for changes in CRC screening based on the time period relative to the COVID‐19 pandemic. Multivariable testing was used to adjust for confounders in models that included age, biologic sex, race, sponsor rank, beneficiary category, and service branch. In line with prior research,^18^, ^19^ missing race was handled using imputation with reweighted estimating equations in all adjusted analyses. In this setting, enrollees with missing race were excluded, and reweighted estimating equations were applied to adjust the remaining data. This is a previously validated technique that has been suggested as the acceptable approach to address missing race data in military healthcare registries.^18^, ^19^

In secondary testing, we assessed the interaction between CRC screening and race and sponsor rank in the time periods relative to the COVID‐19 pandemic. Statistical significance was defined, a priori, as variables with an odds ratio (OR) and 95% confidence interval (CI) exclusive of 1.0 with a *p* < 0.05. This research was found exempt by the Institutional Review Board of the Uniformed Services University of the Health Sciences. All statistical analyses were performed using SAS v9.4 (SAS Institute).

## 3 RESULTS

During the study period, we identified 1,749,688 eligible individuals. Among these, 556,649 (32%) underwent CRC screening (Figure 1). There were 288,492 screenings performed in the pre‐COVID‐19 period and 268,156 in the COVID‐19 period. There were differences in the sociodemographic composition of the cohorts based on time periods that were statistically significant given the size of our sample (Table 1). In terms of the eligible population as a whole, all modes of testing were diminished between the two time frames, with essentially no meaningful change in the rate of Cologuard DNA testing (4% in both epochs). Following the onset of the COVID‐19 pandemic, CRC screening overall decreased from 34% in the pre‐pandemic period to 30% following the onset of the pandemic (*p* < 0.001). This finding persisted even after adjusting for confounders in multivariable analysis (OR for the pandemic timeframe: 0.79; 95% CI: 0.27, 0.31; *p* < 0.001; Table 2). Among those receiving testing, the use of colonoscopy significantly decreased from the pre‐COVID‐19 period (65%) to the COVID‐19 period (62%; *p* < 0.001), while the use of fecal occult blood testing (22% vs. 24%; *p* < 0.001) and Cologuard DNA testing (12% vs. 13%; *p* < 0.001) both significantly increased. When compared to colonoscopy as the referent, the odds of receiving fecal occult blood testing increased by 19% in the COVID‐19 period (OR: 1.19; 95% CI: 1.17, 1.21), while that of Cologuard DNA testing increased by 21% (OR: 1.21; 95% CI: 1.19, 1.24).

**TABLE 1:** Baseline characteristics.

	All encounters	Pre‐COVID‐19 period	COVID‐19 period	*p*‐value
	*N* = 1,749,688	*N* = 852,986	*N* = 896,702	
	*N* (%)	*N* (%)	*N* (%)	
Sex				
Female	873,081 (49.90)	425,243 (49.85)	447,838 (49.94)	Reference
Male	876,590 (50.10)	427,734 (50.15)	448,856 (50.06)	0.238
Age group				
45–49	613,708 (35.08)	276,878 (32.46)	336,830 (37.56)	<0.001
50–54	404,510 (23.12)	206,799 (24.24)	197,711 (22.05)	<0.001
55–59	391,864 (22.40)	200,102 (23.46)	191,762 (21.39)	<0.001
60–64	339,606 (19.41)	169,207 (19.84)	170,399 (19.00)	Reference
Race				
White	727,645 (41.59)	354,354 (41.54)	373,291 (41.63)	Reference
Black	260,559 (14.89)	126,866 (14.87)	133,703 (14.91)	0.925
Other	55,648 (3.18)	26,417 (3.10)	29,231 (3.26)	<0.001
Asian/Pacific Islander	81,174 (4.64)	38,794 (4.55)	42,380 (4.73)	<0.001
American Indian/Alaska Native	9253 (0.53)	4373 (0.51)	4880 (0.54)	0.006
Missing	615,399 (35.17)	302,182 (35.43)	313,217 (34.93)	<0.001
Beneficiary category				
Dependent	814,386 (46.55)	393,394 (46.12)	420,992 (46.95)	<0.001
Retiree	799,458 (45.69)	398,524 (46.72)	400,934 (44.71)	<0.001
Active duty	135,289 (7.73)	60,815 (7.13)	74,474 (8.31)	Reference
Other	538 (0.03)	245 (0.03)	293 (0.03)	0.787
Service				
Army	648,979 (37.09)	313,546 (36.76)	335,433 (37.41)	Reference
Air Force	521,445 (29.80)	257,813 (30.22)	263,632 (29.40)	<0.001
Navy	425,977 (22.90)	207,478 (23.12)	218,499 (22.70)	<0.001
Marines	113,901 (6.51)	54,601 (6.40)	59,300 (6.61)	0.019
Other	48,056 (2.75)	22,875 (2.68)	25,181 (2.81)	0.003
Rank				
Senior Enlisted	1,262,607 (72.17)	617,586 (72.41)	645,021 (71.94)	0.653
Senior Officer	257,393 (14.71)	126,025 (14.78)	131,368 (14.65)	Reference
Junior Officer	207,964 (11.89)	99,387 (11.65)	108,577 (12.11)	<0.001
Junior Enlisted	21,567 (1.23)	9903 (1.16)	11,664 (1.30)	<0.001
Sector (*n* = 556,649)				
Private	389,292 (69.93)	202,820 (70.30)	186,472 (69.54)	<0.001
Direct	167,357 (30.07)	85,673 (29.70)	81,684 (30.46)	Reference
CRC Screening overall	556,649 (31.81)	288,493 (33.82)	268,156 (29.90)	<0.001
Colonoscopy	353,227 (20.19)	188,283 (22.07)	164,944 (18.39)	Reference
Sigmoidoscopy	1180 (0.07)	635 (0.07)	545 (0.06)	0.727
FOBT testing	126,895 (7.25)	62,793 (7.36)	64,102 (7.15)	<0.001
Cologuard DNA testing	70,393 (4.02)	34,276 (4.02)	36,117 (4.03)	<0.001
CT scan	4954 (0.28)	2506 (0.29)	2448 (0.27)	<0.001

**TABLE 2:** Factors associated with colorectal cancer screening for the cohort as a whole.

	CRC Screening No	CRC Screening Yes	Multivariable analysis Odds ratio (95% CI)	*p*‐value
	*N* = 1,193,039	*N* = 556,649	*N* = 1,134,289	
	*N* (%)	*N* (%)		
Sex				
Female	584,537 (49.00)	288,544 (51.84)		Reference
Male	608,486 (51.00)	268,104 (48.16)	0.97 (0.96–0.99)	<0.001
Age group				
45–49	430,166 (36.06)	183,542 (32.97)	1.09 (1.07–1.11)	<0.001
50–54	260,403 (21.83)	144,107 (25.89)	1.44 (1.42–1.46)	<0.001
55–59	254,074 (21.30)	137,790 (24.75)	1.48 (1.46–1.50)	<0.001
60–64	248,396 (20.82)	91,210 (16.39)	Reference	–
Race				
White	494,314 (41.43)	233,331 (41.92)	–	Reference
Black	178,836 (14.99)	81,733 (14.68)	1.07 (1.05–1.08)	<0.001
Other	36,933 (3.10)	18,715 (3.36)	1.08 (1.06–1.10)	<0.001
Asian/Pacific Islander	56,818 (4.76)	24,356 (4.38)	0.92 (0.90–0.93)	<0.001
American Indian/Alaska Native	6685 (0.56)	2568 (0.46)	0.81 (0.76–0.86)	<0.001
Missing	419,453 (35.16)	195,946 (35.20)	–	–
Beneficiary category				
Active duty	79,086 (6.63)	56,203 (10.10)	–	Reference
Dependent	547,425 (45.89)	266,961 (47.96)	0.70 (0.69–0.71)	<0.001
Retiree	566,071 (47.45)	233,387 (41.93)	0.63 (0.62–0.64)	<0.001
Other	448 (0.04)	90 (0.02)	0.21 (0.14–0.30)	<0.001
Service				
Army	445,558 (37.35)	203,421 (36.54)	–	Reference
Air Force	355,374 (29.79)	166,071 (29.83)	1.00 (0.99–1.02)	0.202
Navy	283,340 (23.75)	133,942 (24.06)	0.99 (0.97–1.00)	<0.001
Marines	78,916 (6.61)	34,985 (6.28)	0.87 (0.85–0.89)	<0.001
Other	29,827 (2.50)	18,229 (3.27)	1.12 (1.08–1.15)	<0.001
Rank				
Senior Officer	883,853 (74.09)	378,754 (68.05)	–	Reference
Senior Enlisted	155,327 (13.02)	102,066 (18.34)	0.62 (0.61–0.63)	<0.001
Junior Officer	136,850 (11.47)	71,114 (12.78)	0.81 (0.79–0.83)	<0.001
Junior Enlisted	16,891 (1.42)	4676 (0.84)	0.28 (0.27–0.31)	<0.001
COVID‐19	628,546 (52.68)	268,156 (48.17)	0.79 (0.78–0.80)	<0.001

In our secondary analyses, we found that prior to the COVID‐19 pandemic, Asian/Pacific Islanders (OR: 0.94; 95% CI: 0.92, 0.96) and American Indian/Alaskan natives (OR: 0.83; 95% CI: 0.78, 0.89) were less likely to receive CRC screening as compared to Whites (Table 3). Black beneficiaries had a slightly higher likelihood of undergoing screening (OR: 1.02; 95% CI: 1.00, 1.03) compared to Whites. In the COVID‐19 period, Black beneficiaries demonstrated lower odds of undergoing colorectal screening (OR: 0.87; 95% CI: 10.86, 0.89), while those of Asian/Pacific Islanders (OR: 0.76; 95% CI: 0.75, 0.78) and American Indians/Alaskan Natives were also lower (OR: 0.64; 95% CI: 0.60, 0.69).

**TABLE 3:** Factors associated with colorectal cancer screening assessing the interaction between race and COVID‐19 period.

	Multivariable analysis Odds ratio (95% CI)	*p*‐value
	*N* = 1,134,289	
Sex		
Female		Reference
Male	0.91 (0.90–0.92)	<0.001
Age group		
45–49	1.04 (1.03–1.05)	<0.001
50–54	1.39 (1.37–1.41)	<0.001
55–59	1.43 (1.41–1.45)	<0.001
60–64	Reference	–
Race		
White pre‐COVID‐19	–	Reference
Black pre‐COVID‐19	1.02 (1.00–1.03)	0.014
Asian/Pacific Islander pre‐COVID‐19	0.94 (0.92–0.96)	<0.001
American Indian/Alaska Native pre‐COVID‐19	0.83 (0.78–0.89)	<0.001
Other pre‐COVID‐19	1.11 (1.08–1.14)	<0.001
White and COVID‐19	0.81 (0.80–0.82)	<0.001
Black and COVID‐19	0.87 (0.86–0.89)	<0.001
Asian/Pacific Islander and COVID‐19	0.76 (0.75–0.78)	<0.001
American Indian/Alaska Native and COVID‐19	0.64 (0.60–0.69)	<0.001
Other and COVID‐19	0.88 (0.86–0.90)	<0.001
Beneficiary category		
Active duty	–	Reference
Dependent	0.75 (0.74–0.76)	<0.001
Retiree	0.61 (0.60–0.62)	<0.001
Other	1.20 (1.17–1.23)	<0.001
Service		
Army	–	Reference
Air Force	1.06 (1.05–1.07)	<0.001
Navy	1.06 (1.05–1.07)	<0.001
Marines	0.97 (0.96–0.99)	0.002
Other	1.20 (1.17–1.23)	<0.001
Rank		
Senior Officer	–	Reference
Senior Enlisted	0.66 (0.65–0.67)	<0.001
Junior Officer	0.83 (0.82–0.84)	<0.001
Junior Enlisted	0.36 (0.34–0.38)	<0.001

In the setting of sponsor rank, our proxy for socioeconomic status, prior to the pandemic, both Senior Enlisted (OR: 0.66; 95% CI: 0.65, 0.67) and Junior Enlisted (OR: 0.40; 95% CI: 0.36, 0.43) were less likely to undergo CRC screening as compared to Senior Officers. These findings persisted in the pandemic period, with the odds of individuals from both Senior Enlisted (OR: 0.55; 95% CI: 0.54, 0.56) and Junior Enlisted sponsor ranks (OR: 0.27; 95% CI: 0.25, 0.30; Table 4) diminishing as compared to Senior Officers.

**TABLE 4:** Factors associated with colorectal cancer screening assessing the interaction between sponsor rank (our proxy for socioeconomic status) and COVID‐19 period.

	Multivariable analysis × Odds ratio (95% CI)	*p*‐value
	*N* = 1,134,289	
Sex		
Female		Reference
Male	0.91 (0.90–0.92)	<0.001
Age group		
45–49	1.04 (1.03–1.05)	<0.001
50–54	1.39 (1.37–1.41)	<0.001
55–59	1.43 (1.41–1.45)	<0.001
60–64	Reference	–
Race		
White	–	Reference
Black	1.07 (1.05–1.08)	<0.001
Other	1.10 (1.08–1.12)	<0.001
Asian/Pacific Islander	0.94 (0.92–0.95)	<0.001
American Indian/Alaska Native	0.81 (0.77–0.85)	<0.001
Beneficiary category		
Active duty	–	Reference
Dependent	0.75 (0.74–0.76)	<0.001
Retiree	0.61 (0.60–0.62)	<0.001
Other	0.22 (0.15–0.31)	<0.001
Service		
Army	–	Reference
Air Force	1.06 (1.05–1.07)	<0.001
Navy	1.06 (1.05–1.07)	<0.001
Marines	0.97 (0.96–0.99)	0.002
Other	1.19 (1.17–1.23)	<0.001
Rank		
Senior Officer pre‐COVID‐19	–	Reference
Senior Enlisted pre‐COVID‐19	0.66 (0.65–0.67)	<0.001
Junior Officer pre‐COVID‐19	0.85 (0.83–0.87)	<0.001
Junior Enlisted pre COVID‐19	0.40 (0.36–0.43)	<0.001
Senior Officer and COVID‐19	0.83 (0.81–0.85)	<0.001
Senior Enlisted and COVID‐19	0.55 (0.54–0.56)	<0.001
Junior Officer and COVID‐19	0.68 (0.66–0.69)	<0.001
Junior Enlisted and COVID‐19	0.27 (0.25–0.30)	<0.001

## 4 DISCUSSION

In this investigation, we sought to evaluate the impact of the COVID‐19 pandemic on rates of CRC screening among TRICARE beneficiaries in the MHS. While putative details on this topic have been published in the past,^13^, ^14^, ^15^, ^25^ we believed that our use of national healthcare data from a representative US population over a wider timeframe, beyond the initial phase of the pandemic, could provide more comprehensive information that would also enjoy greater generalizability. Further, the stability of the population insured through TRICARE^18^ and lower concerns regarding loss of insurance associated with work restrictions during COVID‐19 may mean that our data provide more robust estimates regarding the impact of the pandemic itself on CRC screening. The results indicate a 21% reduction in the odds of CRC screening in 2020–2021 as compared to pre‐pandemic levels. When accounting for differences in the population of eligible individuals between the pre‐ and post‐pandemic periods, reductions across the board were seen in the use of colonoscopy, sigmoidoscopy, fecal occult testing, and CT screening. Moreover, disparities in receipt of CRC screening among Asians, Pacific Islanders, American Indians, and Alaskan Natives, as well as those of lower SES, either persisted or worsened during the COVID‐19 pandemic.

Our findings are consistent with several other investigations on the impact of COVID‐19 on cancer screening as well as the general utilization of such services among historically underserved populations. For example, researchers from the National Cancer Institute's Population‐based Research to Optimize the Screening Process (PROSPR) reported that CRC screening rates fell by 82% during the first 6 months of 2020 compared to 2019.^25^ In an insured US population, Oakes and colleagues reported a 10%–18% reduction in the use of CRC screening during quarter 32,020 to quarter 42,021, as compared to pre‐pandemic numbers.^15^ Similar figures were encountered for a Japanese cohort,^14^ while a 23% reduction in CRC screening was reported in South Korea.^13^ The 21% reduction in screening encountered in our own data appears consistent with the latter published reports and endorses the external validity of our findings. Similarly, the reduced use of screening among individuals of lower socioeconomic class, Asian, and the Native American population prior to the pandemic is aligned with other reports from this period.^6^, ^7^, ^8^, ^26^

Outside of our determinations regarding reduced colorectal screening overall, the most concerning findings are those that indicate persistent disparities in the use of these services among African Americans, Asians, Pacific Islanders, and Native Americans/Alaskans, as well as individuals of lower socioeconomic backgrounds. Worsening disparities among Native Americans/Alaskans, based on socioeconomic status, were also evident in the pandemic period. In a previous investigation, Perdue et al. had suggested that differences in the use of CRC screening among average‐risk American Indians and Alaskan Natives could be attributed to issues around access and were largely eliminated in the setting of equal access to care in the setting of health maintenance organizations.^8^ As the MHS is a universally insured healthcare system, with several studies substantiating reduced racial healthcare disparities within the covered population,^16^, ^20^, ^22^ the results of our work would appear to contest this claim.

The current findings also countermand reports that enhanced utilization of home‐based tests, such as Cologuard or fecal immunochemical assays, offset the downturn in colonoscopies that occurred over the course of the pandemic. For example, in their study using the NHIS Liu, and Murphy reported that the use of home tests resulted in an overall increase in screening in 2021, particularly among adults over 65, Blacks and Hispanics, and those with lower incomes and educational levels.^27^ These results were not replicated in our investigation. Indeed, there was no demonstrable change in the use of Cologuard testing between the pre‐ and post‐pandemic periods, while all other forms of screening were reduced. The differences between our study and that of Liu and Murphy^27^ may be explained by the fact that our population is under the age of 65 and possesses larger numbers of Asians, American Indians, and Alaskans, which enabled the detection of meaningful differences.

While additional validation in other studies is therefore warranted, given the characteristics of the population insured through TRICARE,^16^, ^17^, ^18^, ^19^, ^20^, ^21^, ^22^ we believe our findings can be assumed to be translatable to the average‐risk US population between ages 50 and 64. At a minimum, the results herald the need for increased outreach to Asians, Native American, and Alaskans, as well as individuals of all races of lower socioeconomic status, to improve uptake of colorectal screening in these groups. There is also a need for increased investigation into the etiologies behind reductions in screening identified among Native Americans/Alaskans and those of lower socioeconomic status and whether these result from attitudes toward healthcare utilization in general, concerns around accessing healthcare in the pandemic environment, vaccine penetrance in these subpopulations, or other related factors. The lack of increased utilization of home tests for colorectal screening might indicate this is an opportunity to explore interest in and promote these more convenient alternatives to colonoscopy, in the overall covered population as well as in the subgroups that demonstrated significant disparities in screening. Experience in the VA has heralded the feasibility of institutional programs that use mailed fecal immunochemical testing to engage eligible patients.^28^ The program is maintained to reach a greater number of eligible individuals, including those who did not seek in‐person primary care, and reduce demand for colonoscopies, which may be advantageous given the backlogs that have resulted from the COVID‐19 shutdowns.

There are several limitations that should be recognized. Foremost, this remains a retrospective investigation subject to the drawbacks associated with such study designs and the reliance on healthcare claims as the main substrate. Next, our research remains restricted in its scope to the pandemic period of April 2020–September 2021 and cannot assess behaviors or changes that occurred beyond this time window. In this regard, it is important to note that this is the most recent data available from the MHS at the time of this writing. As noted already, given the fact that the TRICARE population has only been found to be representative of the US population under age 64,^18^, ^19^, ^20^, ^21^ our results should not be extrapolated to those over 65 or individuals insured through Medicare. Patterns of utilization and approaches to colorectal screening among those 65 and older may be different from the cohort we studied here. We cannot rule out the possibility that small numbers of patients may have elected to receive screening covered by other insurance products or the VA. However, given prior work performed in this arena,^18^, ^19^, ^20^, ^21^ we do not anticipate that this small cohort of individuals would have an impact on our findings. Finally, some may consider the direct or indirect military affiliation of the covered population a limitation in terms of generalizing to the broader US demographic. We do not believe this is the case, as numerous prior investigations have substantiated the representative nature of the TRICARE population in terms of sociodemographic and clinical variation compared to US civilian cohorts aged 64 and younger.^16^, ^17^, ^18^, ^19^, ^20^, ^21^, ^22^ In addition, we wish to emphasize that only 8% of the individuals under study in this analysis were on active duty. Nonetheless, further validation of our findings in other independent populations with similar clinical and demographic variation would be necessary before definitive investment in our determinations is possible.

In conclusion, in this large study examining changes in the use of colorectal screening between the pre‐ and COVID‐19 pandemic periods, we found a 21% reduction in the odds of screening overall. Reductions in colonoscopies and other types of screening tests were not offset by changes in the use of at‐home tests such as Cologuard. Pre‐pandemic disparities appreciated among Asians, Pacific Islanders, American Indians, Alaskan Natives, and those of lower socioeconomic backgrounds persisted in the pandemic timeframe and even worsened for American Indians/Alaskan Natives, and individuals from lower SES. Our findings suggest several avenues for further research into the drivers of these disparities as well as opportunities for improvement. These could include increased outreach and focused initiatives designed to increase utilization of colorectal screening in at‐risk subgroups as well as enhanced promotion of at‐home fecal immunochemical, or DNA testing.

## AUTHOR CONTRIBUTIONS

**Satish Munigala:** Conceptualization (equal); data curation (equal); formal analysis (equal); methodology (equal); writing – original draft (equal); writing – review and editing (equal). **Andrew J. Schoenfeld:** Conceptualization (equal); methodology (equal); writing – original draft (equal); writing – review and editing (equal). **Vivitha Mani:** Conceptualization (equal); writing – review and editing (equal). **Amanda Banaag:** Conceptualization (equal); methodology (equal); writing – review and editing (equal). **Ada Umoh:** Writing – review and editing (equal). **Christian L. Coles:** Conceptualization (equal); methodology (equal); writing – original draft (equal); writing – review and editing (equal). **Tracey Perez Koehlmoos:** Conceptualization (equal); methodology (equal); resources (equal); supervision (equal); writing – original draft (equal); writing – review and editing (equal).

## CONFLICT OF INTEREST STATEMENT

The contents, views, or opinions expressed in this manuscript are those of the author(s) and do not necessarily reflect the official policy or position of the Uniformed Services University of the Health Sciences, the Department of Defense, or the Departments of the Army, Navy, or Air Force, or the Henry M. Jackson Foundation for the Advancement of Military Medicine, Inc. Mention of trade names, commercial products, or organizations does not imply endorsement by the US Government. The authors declare they have no conflicts of interest.

## Supporting information


Table S1.

## Data Availability

The data contains personal identifiers and are not publicly available. The data that support the findings of this study are available on request from the corresponding author.
