# Supplementary material for: Disparities in the use of colorectal cancer screening in a universally insured population during the COVID‐19 pandemic
Source: Cancer Med. 2023 Aug 29;12(17):18201–10. doi: 10.1002/cam4.6400 (PMC10524012; doi:10.1002/cam4.6400)
Supplement: Supplementary file 1 — Table S1. [file CAM4-12-18201-s001.docx]

Supplementary Table 1.

| Colorectal cancer screening | ICD 9/ 10 codes |
| --- | --- |
| Colonoscopy | '45378','45379','45380','45381','45382','45383','45384','45385','45386','45387','45388','45389','45390','45391','45392','45393','45398','G0121' |
| Sigmoidoscopy | '45330','45331','45332','45333','45334','45335','45337','45338','45340', '45341','45352','45346','45347','45349','45350','G0104','G0106' |
| Fecal Occult Blood testing | '82270','82274','G0107','G0328' |
| Cologuard DNA testing | '81528' |
| CT scan | '74261','74262','74263' |
| Colorectal cancer exclusions |  |
| Malignant neoplasm of cecum, Malignant neoplasm of rectosigmoid junction, Malignant neoplasm of rectum, Malignant neoplasm of anus, unspecified; Secondary malignant neoplasm of large intestine and rectum, Crohn's disease of small intestine without complications, Ulcerative (chronic) pancolitis without complications, Gastroenteritis and colitis due to radiation, Toxic gastroenteritis and colitis, Other specified noninfective gastroenteritis and colitis, Noninfective gastroenteritis and colitis, unspecified; Diverticulitis of large intestine with perforation and abscess without bleeding, Anal polyp, Rectal polyp, Ulcer of anus and rectum, Ulcer of intestine, Polyp of colon, Family history of malignant neoplasm of digestive organs, Family history of colonic polyps, Personal history of malignant neoplasm of unspecified digestive organ, Personal history of other malignant neoplasm of large intestine, Personal history of other malignant neoplasm of rectum, rectosigmoid junction, and anus; Personal history of colonic polyps, diagnosis or past history of total colectomy or colorectal cancer | '153','154','1541','1975','555','556','5581','5582','5589','562','569','56941','56982','2113',  'V160','V1851','V1000','V1005','V1006','V1272'  'C18', 'C19', 'C20','C21', 'C785','K50','K51','K520','K521','K5289','K57','K620',  'K626','K633','K635','Z800','Z8371','Z8500','Z85038','Z85048','Z86010'  'G0213','G0214','G0231','G9711','44150','44151','44152','44153','44155','44156','44157','44158', '44210','44211','44212' |
